# A global dataset of spatiotemporal co-occurrence patterns of avian influenza virus-associated migratory birds

**DOI:** 10.1038/s41597-026-06701-w

**Published:** 2026-02-03

**Authors:** Jun Ma, Yan-He Wang, Yun-Bo Qiu, Jin-Jin Chen, Yun Han, Yan Zhang, Sheng-Hong Lin, Qing-Jie Wang, Long-Tao Chen, Xin-Jing Zhao, Sheng Zhang, Tian Tang, Yao Tian, Yu-Feng Yang, Qiang Xu, Zi-Da Meng, Chen-Long Lv, Guo-Lin Wang, Feng Hong, Li-Qun Fang

**Affiliations:** 1 School of Public Health, the Key Laboratory of Environmental Pollution Monitoring and Disease Control, Ministry of Education, Guizhou Medical University, Guiyang, 561113 P. R. China; 2 State Key Laboratory of Pathogen and Biosecurity, Academy of Military Medical Science, Beijing, 100071 P. R. China; 3 The 968th Hospital of Joint Logistics Support Force of PLA, Jinzhou, Liaoning P. R. China; 4 Department of Clinical Laboratory, the Second Affiliated Hospital of Anhui Medical University, Hefei, P. R. China

**Keywords:** Animal migration, Behavioural ecology

## Abstract

Migratory birds facilitate the cross-regional spread of pathogens such as avian influenza virus (AIV). Interspecies interactions among multiple migratory bird species within shared spatiotemporal habitats can substantially enhance pathogen transmission and evolution, thereby posing potential risks to public health and livestock safety. Recent advances in tracking technologies, such as GPS, combined with publicly accessible databases like Movebank, have enabled the reconstruction of avian migratory pathways. However, existing tracking data are largely collected from individual species, remain species-specific and are insufficient for characterizing interspecies contact during migration. By integrating available tracking data from 62 migratory bird species (comprising 3,944 individual records), this study constructed a co-occurrence dataset comprising 50 migratory bird species that exhibited spatial and temporal overlap at shared locations, with a daily temporal resolution and spatial resolution aligned with first-level administrative divisions. This dataset can facilitate the identification of potential hotspots for migratory bird-associated pathogen evolution, thereby providing data-driven support for the prevention and control of emerging infectious diseases.

## Background & Summary

The migration of birds represents one of the most remarkable ecological phenomena in nature. Each year, thousands of migratory birds traverse continents and oceans to undertake regular migrations between their breeding and wintering grounds^1,2^. This behavior serves as a crucial survival and reproduction strategy for migratory birds while playing an essential role in sustaining the balance of global ecosystems and preserving biodiversity^3–5^. Nevertheless, amid escalating global ecological and environmental changes, the risk of pathogen transmission during bird migration has substantially increased, rendering migratory birds a significant pathway for cross-regional pathogen transmission^6–8^.

Migratory birds, while traversing multiple ecosystems and crossing international borders during their migration, may serve as vectors for pathogens between breeding sites, stopover sites, and wintering grounds. This poses a potential threat to both public health and ecosystem health. Recent studies have revealed the patterns of specific pathogen carriage and transmission by migratory birds along their migratory routes^9–13^. For instance, continuous surveillance by Wang *et al*. on wild waterfowl has shown that these birds can shed a variety of pathogens, including avian influenza viruses and reoviruses, during migration^14^. Such pathogens pose a significant transmission risk not only along migratory corridors but also within the habitats frequented by migratory birds, such as wetlands, agricultural areas, and artificial water bodies^15–27^. High-density bird aggregations in these areas increase contact rates among bird individuals, thereby facilitating pathogen transmission^28,29^. Concurrently, aquatic environments enable prolonged pathogen survival, allowing infectious agents to remain viable for extended periods in water^30,31^. Additionally, interactions between migratory birds and domestic poultry at wintering and stopover sites may facilitate cross-species transmission of pathogens through direct contact in shared aquatic environments and feeding areas^32,33^, thereby facilitating pathogen exchange, viral spillover, and potential adaptation within domestic populations^34,35^. Among these pathogens, avian influenza virus (AIV) is particularly noteworthy, as its global dissemination is closely associated with the long-distance migration of migratory birds^36–40^. For instance, research has demonstrated that avian influenza viruses (AIVs) in migratory birds wintering within the Yangtze River wetlands of eastern China exhibit rich subtype diversity and complex genomic characteristics^41^. Notably, some viral segments show high genetic similarity to strains currently circulating in migratory bird populations in North America. This pattern suggests that migratory birds may play a critical role in facilitating intercontinental pathogen transmission, as evidenced by successive multi-continental outbreaks of highly pathogenic avian influenza (HPAI), including the 2005‒2006 H5N1 outbreak, the 2014‒2015 emergence of H5N8, and the ongoing H5N1 clade 2.3.4.4b outbreak that began in 2020^42–47^. Building on this evidence, comprehensive studies have integrated avian migration data with AIVs transmission networks. These studies revealed the spatiotemporal dynamics and transmission pathways of the virus across diverse geographical regions^48^. These findings collectively underscore the important role of migratory birds in pathogen transmission and highlight the necessity of implementing robust surveillance and risk management during bird migration.

Advancements in tracking technologies, including Global Positioning System (GPS) and satellite telemetry, have significantly enhanced our understanding of migratory bird movements^49^. Publicly accessible databases like Movebank support the sharing of tracking data, hereby facilitating large-scale analyses of migration patterns^50,51^. However, current research has largely concentrated on single-species studies, resulting in fragmented datasets that hinder the identification of spatiotemporal overlap and potential interspecies interactions during migration. This limitation might impede a comprehensive assessment of cross-species pathogen transmission risks. Integrating tracking data from multiple species and applying standardized analytical methods can address this gap. Density-based clustering algorithms, such as T-DBSCAN, have been increasingly applied to migratory bird tracking data^52,53^ and provide a standardized approach for identifying stopover sites among different species. These approaches enable the detection of stopover sites from tracking data, thereby facilitating multi-species spatiotemporal analyses when utilized in integrated datasets.

This study aims to integrate existing migratory bird tracking data from the Movebank database and apply the T-DBSCAN algorithm to identify specific spatiotemporal locations of various migratory bird species on given dates. A spatiotemporal co-occurrence dataset of migratory birds was established with a daily temporal resolution and spatial resolution aligned with first-level administrative divisions. Co-occurrence analyses were conducted for migratory bird species with documented detections of AIVs to illustrate the role of this dataset in untangling avian influenza virus variation hotspots and identifying relevant key migratory bird species. This provides a theoretical foundation and data support for the prevention and control of emerging and re-emerging infectious diseases.

## Methods

### Data collection

To collect migration data for bird species with documented detections of AIVs, we obtained relevant tracking data from Movebank, a global database dedicated to bird movement research. The selection of migratory bird species was based on a published systematic review^54^, which identified 175 migratory bird species with documented natural AIV infections. These species account for infection records across 99 distinct AIV subtypes, providing a comprehensive species list and evidence base for this study.

Based on the taxonomic information, including order, family, genus, species, and common name, for the 175 migratory bird species, we systematically retrieved all

available tracking dataset from the Movebank platform. During data acquisition, only research projects with appropriate data use licenses were included to ensure compliance with relevant data-sharing agreements and terms of use for all downloaded and utilized data. No time restrictions were applied during the retrieval process, thereby maximizing the inclusion of data representing diverse migration patterns and geographic regions.

Through systematic retrieval, tracking data from 182 independent studies involving 62 species were preliminarily obtained from the Movebank platform. Detailed AIV subtype associations for these 62 species, including detection records and corresponding references, are provided in “AIV associated species list.xlsx” available via figshare^55^. All collected raw data contained detailed spatiotemporal records, with metadata such as geographic coordinates, timestamps, individual identification codes, tracking device types, and research project information. The tracking technologies employed in these datasets include GPS positioning, Argos Doppler-based positioning, radio transmitters, solar-powered geolocators, and Sigfox geolocation systems.

**Fig. 1:**
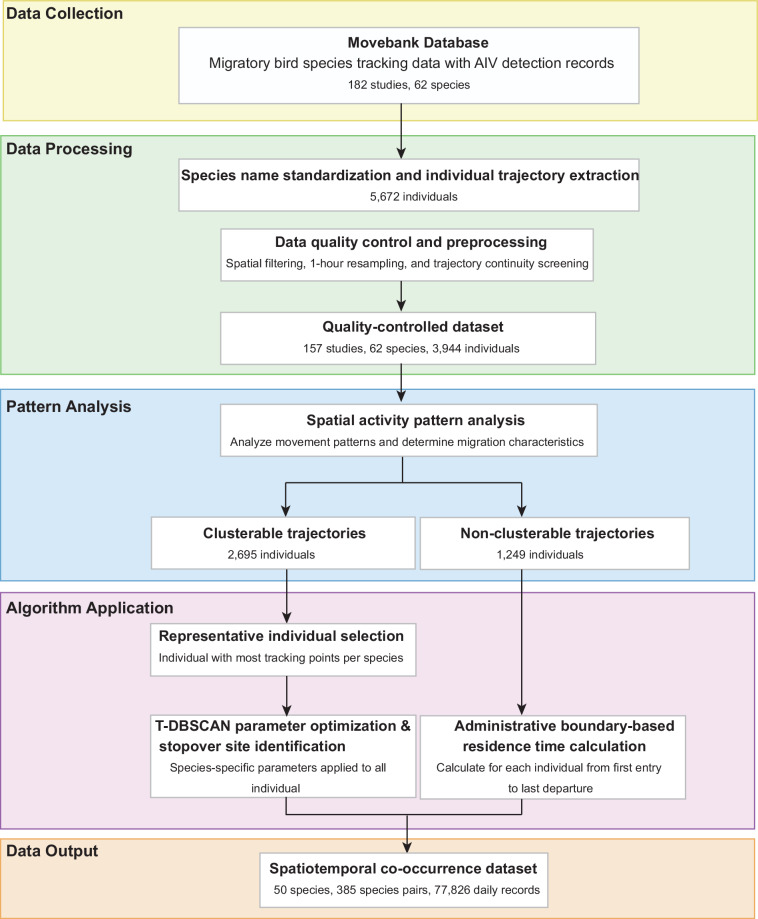
Workflow for constructing the spatiotemporal co-occurrence dataset of migratory birds.

**Data processing**. This study established a comprehensive workflow for identifying stopover sites of migratory birds during their migration (Fig. 1), including data collection, preprocessing, migration pattern identification, representative individual selection, and stopover site identification. Raw tracking data were organized by research project. Individual trajectories within each study were separated based on the “individual-local-identifier” field, and species identification was assigned according to the scientific names recorded in the “taxon-canonic-name” field. To address inconsistencies arising from the mixed use of scientific and common names in the database, all species names were standardized according to the taxonomy of the Handbook of the Birds of the World and BirdLife International^56^. Following the processing of individual trajectory separation and species identification, tracking data for a total of 5,672 individuals were compiled.

To focus the analysis on terrestrial ecosystems, a spatial intersection analysis was conducted using the sf package in R^57^. Specifically, terrestrial boundary data at the first administrative level from the Global Administrative Areas database (GADM, https://www.gadm.org) were loaded to spatially filter the tracking points: only those located within terrestrial administrative polygons were retained, whereas points falling within marine areas were excluded. To reduce redundancy in high-frequency tracking data, the original dataset was down-sampled to a uniform temporal resolution of 1-hour intervals. To ensure continuity in individual trajectory analysis, only individuals meeting the following criteria were retained: after down-sampling, at least 80% of consecutive points in their valid tracking sequences exhibited time intervals not exceeding 24 hours. It is noteworthy that these criteria were applied at the individual level. An individual was retained for further analysis only if its overall trajectory met the quality standards; otherwise, it was entirely excluded. Ultimately, valid tracking data from 3,944 individuals across 62 species, derived from 157 studies^58–214^, were retained for subsequent identification of stopover sites (detailed information is provided in “Individual tracking data.xlsx” via figshare^55^).

The identification of migratory bird stopover sites was conducted using an adaptive algorithmic strategy. First, a visual analysis of individual movement trajectories was performed to assess their suitability for cluster analysis based on spatiotemporal distribution patterns. Suitability criteria were defined as follows: trajectories considered appropriate for clustering exhibited distinct directional and continuous movement patterns, characterized by regular displacements along relatively fixed directions, with tracking points forming recognizable aggregation patterns in space; trajectories unsuitable for clustering primarily displayed overly sparse tracking points, irregular flight paths (e.g., diamond-shaped patterns), or lacked clear spatial aggregation characteristics. Based on this assessment, trajectories from 2,695 individuals were determined to be applicable for cluster analysis, while trajectories from 1,249 individuals were excluded due to sparse data or irregular patterns.

For these suitable trajectories, we employed the T-DBSCAN algorithm^52,53^ to identify discrete stopover sites from individual migration trajectories. This spatiotemporal clustering method was selected for its ability to directly output discrete stopover events based on explicit temporal constraints (minStayTime and maxIntervalTime)^52^, allowing operational definitions aligned with species-specific movement ecology. For individuals suitable for clustering, the individual with the highest number of tracking points within each species was selected as the representative for parameter optimization of the T-DBSCAN algorithm. The parameter optimization procedure was conducted as follows: The leaflet package in R was used to generate annual interactive migration trajectory maps for the selected individuals. Through visual inspection, the stopover region with the largest activity area in each year was identified. The annotation function of the interactive map was utilized to determine the start and end point indices for each stopover region. All coordinates (longitude and latitude) within this index range were used as input, and the Welzl algorithm^215^ was applied to calculate the minimum enclosing circle radius (in kilometers) encompassing all these points. Following cross-annual analysis of all stopover regions for the representative individuals of each species, the calculated radii were used as the baseline value for the species’ spatial radius parameter (eps), reflecting its typical activity range. In parallel, local recurrent movement regions within the representative individual’s trajectory were identified via visual inspection of the interactive maps. Such trajectory segments were required to contain at least three consecutive tracking points and exhibit a back-and-forth movement pattern within a specific area. The temporal information for each point in these segments was obtained using the map’s annotation function, and the time span (in hours) of these recurrent movement segments was calculated. By analyzing the distribution of these time spans, the minimum value was recorded as the baseline for the species’ minimum stay time parameter (minStayTime).

Following parameter optimization validation (see Technical Validation section), the final T-DBSCAN parameters were set as follows: eps was set to 1.2 times the baseline value (providing optimal spatial buffering), minStayTime was set to 0.8 times the baseline value, and maxIntervalTime was set to 60 days. The optimized T-DBSCAN spatiotemporal clustering algorithm was applied to all migratory individuals suitable for clustering to identify stopover sites. For individuals not suitable for clustering, the residence time within each first-level administrative division was calculated based on the corresponding boundary data, defined as the time span from the first recorded entry to the last recorded exit from that division.

## Data Records

The final constructed spatiotemporal co-occurrence dataset is provided as three separate interrelated files, publicly available via figshare^55^. The database includes the following fields:

### Daily species pairs.xlsx

This worksheet constitutes the core component of the dataset, documenting co-occurrence events for specific species pairs within specific first-level administrative divisions on specific dates. Key fields include:

1. **Countries:** Countries where co-occurrence events were recorded.
2. **First-level administrative division:** The first-level administrative division where co-occurrence events were recorded.
3. **Date:** The date of the co-occurrence event (recorded by month-day).
4. **Species 1:** The scientific name of the first species in the species pair.
5. **Species 2:** The scientific name of the second species in the species pair.
6. **Daily species count:** The total number of species recorded in the first-level administrative division on that day.
7. **Pair name:** The standardized name of the species pair.

### Administrative division summary.xlsx

This worksheet provides summary statistics for each first-level administrative division:

1. **Countries:** Countries where co-occurrence events were recorded.
2. **First-level administrative division:** The first-level administrative division where co-occurrence events were recorded.
3. **Total pair-days:** The total number of species pair-days recorded within the first-level administrative division. A pair-day is defined as the number of unique species pairs co-occurring within the same first-level administrative division on a given calendar date (recorded as month-day), calculated using the combination formula C(n,2), where n represents the number of species observed on that date.
4. **Co-occurrence days:** The number of distinct dates (recorded by month-day) on which multi-species co-occurrence was recorded.
5. **Max daily pairs:** The maximum number of species pairs recorded on any single co-occurrence date (recorded by month-day) within that first-level administrative division across all recorded dates.
6. **Total species involved:** The total number of distinct species involved.
7. **Unique pair types:** Number of unique species pair types.

### Species pair summary.xlsx

1. **Pair name:** Standardized name of the species pair.
2. **Species 1:** Scientific name of the first species in the pair.
3. **Species 2:** Scientific name of the second species in the pair.
4. **Countries:** All countries where the co-occurrence of this species pair has been recorded, separated by semicolons.
5. **Total co-occurrence division-days:** For all first-level administrative divisions where the co-occurrence of the species pair was recorded, the cumulative number of division-days was calculated. Division-days are defined as a distinct combination of calendar date (month-day) and first-level administrative division on which the species pair co-occurred.
6. **First-level administrative division count:** The number of first-level administrative divisions where co-occurrence of this species pair occurred.
7. **Date range:** The temporal span of co-occurrence events, showing the month and day of the first and last recorded co-occurrence of the species pair.

(8–19) **January–December:** Monthly distribution of co-occurrence, showing the number of days (recorded by month-day) and percentage for each month.

**Fig. 2:**
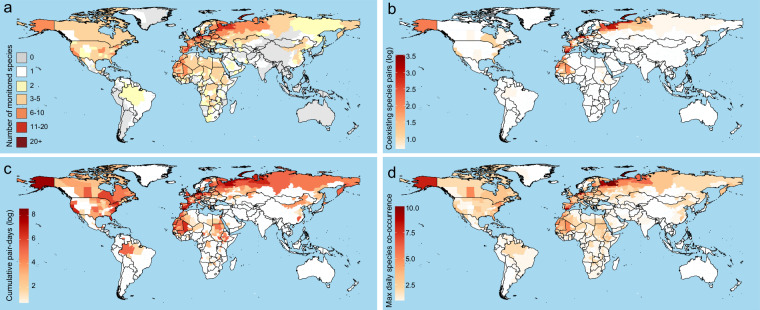
Spatiotemporal distribution patterns of tracking data for migratory bird species with records of AIV detection across first-level administrative divisions. (**a**) Number of species tracked; (**b**) Number of unique species pairs recorded (log-transformed); (**c**) Cumulative pair-days (log-transformed); (**d**) Maximum number of species recorded in a single day. A pair-day is defined as the number of unique species pairs co-occurring within the same first-level administrative division on a given calendar date (recorded as month-day), calculated using the combination formula C(n,2), where n represents the number of species observed on that date.

This dataset documents co-occurrence events among migratory bird species across 488 first-level administrative divisions worldwide. Based on tracking data from 62 migratory bird species with documented AIV detection (derived from 3,944 individuals across 157 studies), the dataset captures spatiotemporal co-occurrence events involving 50 species that demonstrated spatial overlap within the same first-level administrative division and temporal overlap on the same calendar date. A total of 385 unique species pairs are represented across 77,862 daily co-occurrence records. Summary statistics of the first-level administrative divisions presented in the worksheet indicate significant spatial heterogeneity in species richness, co-occurrence intensity, and timing of daily peaks across regions (Fig. 2). The number of species tracked per first-level administrative division ranges from 1 to 21 (Fig. 2a), and the number of species pair combinations exhibits distinct spatial clustering characteristics (Fig. 2b). The distribution of cumulative paired days reflects spatial hotspots of migratory bird activity (Fig. 2c), while the maximum number of species recorded on a single day untangles the spatiotemporal concentration of migratory activities (Fig. 2d).

**Fig. 3:**
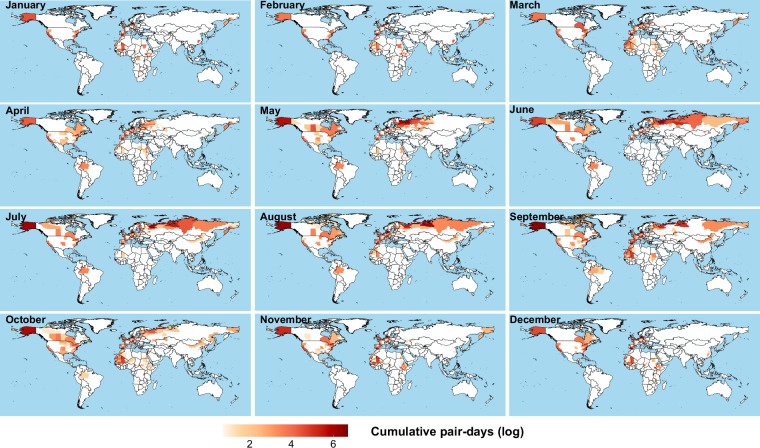
Monthly spatiotemporal distribution patterns of tracking data for migratory bird species with a history of AIV detection. The distribution of cumulative pair-days (log-transformed) per first-level administrative division per month illustrates the seasonal variation in spatiotemporal distribution patterns across different months. A pair-day is defined as the number of unique species pairs co-occurring within the same first-level administrative division on a given calendar date (recorded as month-day), calculated using the combination formula C(n,2), where n represents the number of species observed on that date.

**Fig. 4:**
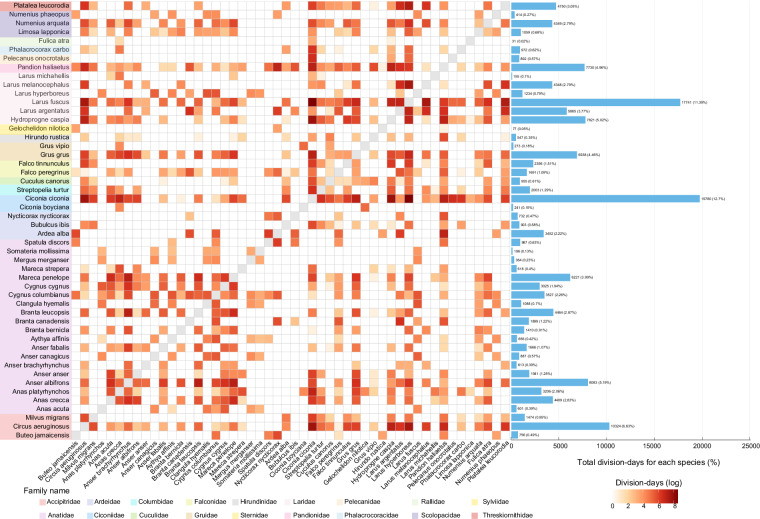
Co-occurrence patterns among migratory bird species with previous records of AIV detection. Each cell in the heatmap matrix represents the logarithm of the number of division-days in which a specific pair of species co-occurred. Species are ordered by taxonomic family. Different taxonomic families are distinguished by colored background regions and family labels at the bottom. The bar plot on the right displays the sum of division-days for each species with all other species and its corresponding proportion. Division-days are defined as distinct combinations of calendar date (month-day) and first-level administrative division on which the species pair co-occurred.

**Fig. 5:**
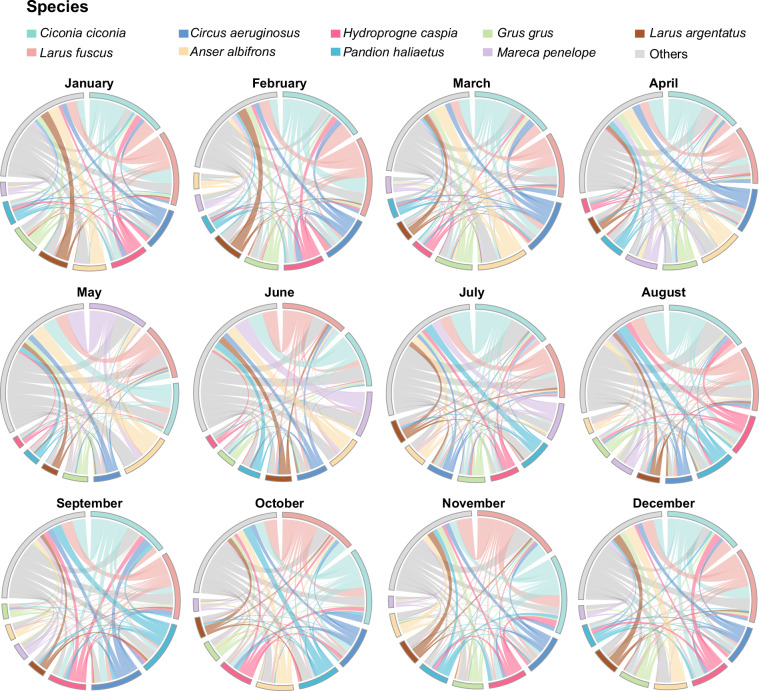
Monthly co-occurrence patterns of migratory bird species with a history of AIV detection. The chord diagram illustrates the spatiotemporal distribution patterns among species each month. The arcs represent species, and the thickness of the connecting chords indicates the number of division-days for each species pair. For each month, the top 9 species with the highest number of division-days are displayed; the remaining species are grouped into “Others” (shown in gray). Division-days are defined as distinct combinations of calendar date (month-day) and first-level administrative division on which the species pair co-occurred.

From a temporal perspective, the 77,862 daily records included in the worksheet demonstrate that species co-occurrence events are not uniformly distributed throughout the year. As shown in Fig. 3, the monthly distribution of cumulative paired days clearly illustrates this inter-monthly variation pattern. The worksheet further summarizes the spatiotemporal statistical characteristics of all unique species pairs, including the number of days each pair was observed, the number of primary administrative divisions covered, and the temporal span, among other information. Figure 4 presents the co-occurrence patterns among species in the form of a heatmap. Figure 5 shows monthly chord diagrams that illustrate how species co-occurrence patterns vary throughout the year.

In addition to the core dataset, the figshare repository includes the following data files: (1) “AIV associated species list.xlsx” details the associations of AIV subtype for the 62 species, including detection records and corresponding references; (2) “Individual tracking data.xlsx” provides detailed tracking information for all 3,944 individuals included in the analysis; (3) “Spatial co-occurrence validation.xlsx” and (4) “Temporal co-occurrence validation.xlsx” respectively contain the validation results of comparing co-occurrence patterns between the known AIV-associated migratory birds (62 species) and the extended migratory bird range (143 species) scenarios in spatial and temporal dimensions; and (5) “Tracking data sources.xlsx” provides the data sources and attribution for all bird tracking datasets used in this study.

## Technical Validation

### Validation of AIV-associated migratory bird range representatives

Given the rapid evolution of AIV and its potential for expansion into new migratory bird species, an analysis of the range extension of AIV-associated migratory birds was conducted to evaluate the robustness of spatiotemporal co-occurrence patterns based on known AIV-associated migratory birds under scenarios of AIV-associated migratory bird range expansion.

Two analytical scenarios were established for comparison: (1) the “Known AIV-associated migratory birds” scenario, based on 62 migratory bird species with documented AIV detection; and (2) the “Extended AIV-associated migratory bird range” scenario, which included all available migratory bird species from the Movebank platform (totaling 143 species from 282^58–214,216–334^ studies). Both datasets were processed using identical data processing pipelines and spatiotemporal co-occurrence definitions.

Spatial pattern consistency: Among the 488 common administrative divisions, the rankings of co-occurrence intensity were highly consistent (Spearman’s *ρ* = 0.80, *p* < 0.05), indicating that the identification of high co-occurrence intensity regions remained stable after AIV-associated migratory bird range expansion.

Temporal Pattern Consistency: The monthly co-occurrence intensity patterns also demonstrated high consistency (Spearman’s *ρ* = 0.82, *p* < 0.05), suggesting that the critical time windows for potential transmission remained consistent under different AIV-associated migratory bird range assumptions.

The strong correlations in both spatial and temporal dimensions (*ρ* ≥ 0.8, *p* < 0.05) indicated that the primary hotspot regions and critical time windows remained stable following host range expansion, thereby validating the representativeness and robustness of risk assessments based on known AIV-associated migratory birds (detailed results of spatial comparison and temporal comparison are provided in “Spatial co-occurrence validation.xlsx” and “Temporal co-occurrence validation.xlsx” respectively, available via figshare^55^).

### Validation of the biological relevance of co-occurrence sites

To validate whether the identified co-occurrence sites correspond to actual interspecies contact or pathogen environmental sharing, viral sequences from co-occurring species pairs collected at identified hotspots were retrieved from the GISAID database and analyzed for genetic similarity. Sequences were aligned using MAFFT^335^, and genetic similarity (based on p-distance) was calculated using R.

In the Netherlands, a co-occurrence hotspot identified by the dataset, the herring gull (*Larus argentatus*) and the lesser black-backed gull (*Larus fuscus*) formed a frequently co-occurring species pair. H5N1 virus strains collected from these two species in April 2022 (accession numbers: EPI_ISL_13201050 and EPI_ISL_12514681) exhibited high genetic similarity across five gene segments (NP: 99.81%, HA: 99.77%, NS: 99.06%, MP: 99.90%, NA: 99.72%). Both samples were collected during the predicted co-occurrence period for these species.

In Belgium, another independent co-occurrence hotspot, H13 virus strains collected from the same species pair in August 2008 (accession numbers: EPI_ISL_63813 and EPI_ISL_63814) demonstrated 99.28% similarity in the HA segment. Sampling occurred within the identified co-occurrence period.

Across two independent geographical regions and time points, the identified co-occurrence hotspots yielded highly similar viral strains ( >99% similarity across gene segments) from co-occurring species pairs. This molecular evidence demonstrates that the identified co-occurrence sites represent not merely statistical spatiotemporal overlap, but correspond to locations where species likely experienced actual pathogen contact or shared contaminated environments. This cross-regional and cross-temporal consistency validates the effectiveness of the tracking data-based co-occurrence identification method in pinpointing key spatiotemporal sites for potential interspecies pathogen transmission.

## Usage Notes

This dataset documents the global spatiotemporal co-occurrence patterns of migratory bird species prior records of AIV detection, offering significant value for epidemiological risks assessment, surveillance optimization, and conservation planning.

### Ecological research applications

The cumulative pair-days and the maximum daily species count presented in “Administrative division summary.xlsx” can be used to analyze the activity intensity of migratory birds and the patterns of species diversity across different regions. The division-days data in “Species pair summary.xlsx” facilitates the identification of spatiotemporal associations between species and potential ecological interactions by quantifying the total number of distinct date-location combinations where species pairs co-occur.

### Disease transmission and variation risk assessment

This dataset can be used to assess the intensity of species contact and assist in estimating the potential risk levels of virus variation across different regions. The “Administrative division summary.xlsx” file provides quantitative metrics for prioritizing surveillance efforts. Divisions with high total pair-days values indicate frequent multi-species interactions, which necessitate enhanced monitoring. Meanwhile, “max daily pairs” values identify areas where numerous species converge simultaneously, suggesting potential transmission hotspots. Surveillance programs should prioritize species pairs that exhibit high-frequency co-occurrence characteristics, focusing on key species combinations where viral strain exchange may occur. The monthly distribution data (columns from January to December) in “Species pair summary.xlsx” facilitates the precise scheduling of surveillance activities, showing exactly when and where peak interspecies interactions are expected in specific administrative divisions.

### Conservation planning applications

The number of species pairs and the total species count within each first-level administrative division can help identify activity hotspots for migratory bird species, thereby providing a scientific basis for the arrangement of disease monitoring sites and the identification of potential high-risk areas. Division-days data can guide the scheduling of monitoring and management activities, facilitating improved disease surveillance during periods when species aggregate with high frequency. Specific applications include: allocating resources to divisions with the highest co-occurrence intensity based on total pair-days metrics, timing monitoring efforts by referring to the monthly peak periods from “Species pair summary.xlsx”, and focusing on species pairs that have demonstrated frequent interactions across multiple administrative regions.

### Data limitations

It should be acknowledged that tracked individuals may not fully represent the entire species populations, as tracking studies often focus on specific age groups or sexes; there may be potential impacts of tracking devices on migratory behavior; individuals with irregular migration patterns may be systematically excluded due to our data filtering criteria (requiring ≥80% of tracking points with time intervals less than 24 hours); T-DBSCAN algorithm parameters were optimized using single representative individuals of each species, which may not fully capture intraspecific behavioral variation; and definitions of co-occurrence are based on administrative boundaries rather than ecological ones. Furthermore, the current AIV-associated wild bird range does not necessarily indicate which other species could potentially be infected by or serve as vectors for AIVs. Essentially, the present state of knowledge primarily reflects a list of species that have either been subjected to experimental infection studies or have had contact with the virus and subsequently exhibited symptoms and/or undergone testing for active infection or serological response, rather than accurately representing the true extent of wild bird species associated with circulating AIV strains.

Additionally, the efforts in migratory bird tracking are distributed unevenly across global regions. This distribution reflects the cumulative spatiotemporal co-occurrence patterns of avian influenza virus-associated migratory birds based on historical research projects, rather than a pre-planned systematic coverage. Our study integrated existing tracking datasets from the Movebank database, within which tracking studies have, historically, predominantly concentrated on certain migratory flyways. These geographic gaps may arise from varying research priorities, species-specific project focuses, along with technological and logistical constraints across regions. Such uneven coverage may lead to an underestimation of co-occurrence patterns in data-sparse areas, such as certain parts of Asia during winter, where sufficient multi-species tracking data are not yet available. It should be recognized that when interpreting the results, the lack of co-occurrence records in specific geographic areas and time periods may be due to data availability rather than the absence of actual interspecies interactions. Nevertheless, our dataset provides a valuable systematic integration of multi-species tracking data and identifies co-occurrence patterns, which represents a comprehensive analysis achievable through current tracking efforts. Disease surveillance applications will yield the greatest effectiveness in well-sampled regions. Meanwhile, the expansion of multi-species tracking in underrepresented areas may uncover additional transmission hotspots that complement our current findings.

## Data Availability

The dataset is publicly available on figshare^55^ at 10.6084/m9.figshare.31044229.v1. It comprises eight Excel files, including three core dataset files and five supporting data files.

## Code availability

The analysis code is publicly accessible at https://github.com/FlowSpatial/Migratory-birds-code.git. It covers trajectory screening, T-DBSCAN-based stopover identification, and spatiotemporal co-occurrence analysis.
